# Circulating Endothelial Cells and Procoagulant Microparticles in Patients with Glioblastoma: Prognostic Value

**DOI:** 10.1371/journal.pone.0069034

**Published:** 2013-07-29

**Authors:** Gaspar Reynés, Virtudes Vila, Tania Fleitas, Edelmiro Reganon, Jaime Font de Mora, María Jordá, Vicenta Martínez-Sales

**Affiliations:** 1 Servicio de Oncología Médica, Hospital Universitari i Politècnic La Fe, Valencia, Spain; 2 Centro de Investigación, Hospital Universitari i Politècnic La Fe, Valencia, Spain; 3 Servicio de Hematología y Oncología Médica, Hospital Clínico Universitario, Valencia, Spain; 4 Instituto de Investigación Sanitaria Hospital La Fe, Valencia, Spain; 5 Servicio de Anatomía Patológica, Hospital Universitari i Politècnic La Fe, Valencia, Spain; Dana-Farber Cancer Institute, United States of America

## Abstract

**Aim:**

Circulating endothelial cells and microparticles are prognostic factors in cancer. However, their prognostic and predictive value in patients with glioblastoma is unclear. The objective of this study was to investigate the potential prognostic value of circulating endothelial cells and microparticles in patients with newly diagnosed glioblastoma treated with standard radiotherapy and concomitant temozolomide. In addition, we have analyzed the methylation status of the *MGMT* promoter.

**Methods:**

Peripheral blood samples were obtained before and at the end of the concomitant treatment. Blood samples from healthy volunteers were also obtained as controls. Endothelial cells were measured by an immunomagnetic technique and immunofluorescence microscopy. Microparticles were quantified by flow cytometry. Microparticle-mediated procoagulant activity was measured by endogen thrombin generation and by phospholipid-dependent clotting time. Methylation status of *MGMT* promoter was determined by multiplex ligation-dependent probe amplification.

**Results:**

Pretreatment levels of circulating endothelial cells and microparticles were higher in patients than in controls (p<0.001). After treatment, levels of microparticles and thrombin generation decreased, and phospholipid-dependent clotting time increased significantly. A high pretreatment endothelial cell count, corresponding to the 99^th^ percentile in controls, was associated with poor overall survival. *MGMT* promoter methylation was present in 27% of tumor samples and was associated to a higher overall survival (66 weeks vs 30 weeks, p<0.004).

**Conclusion:**

Levels of circulating endothelial cells may have prognostic value in patients with glioblastoma.

## Introduction

The current standard of care for newly diagnosed glioblastoma is surgery, radiotherapy, and concomitant daily temozolomide, followed by cycles of this drug given for five days every four weeks. Despite treatment, most patients die within two years of surgery [1]. The proportion of patients who benefit from this therapy is determined only partially by the methylation status of the O6-alkylguanine-DNA-methyltransferase (*MGMT*) gene promoter, which is considered a prognostic factor rather than a predictor of response [2]. Assessment of response in glioblastoma patients is difficult because radiochemotherapy modifies vascular permeability in the tumor area. This alteration can lead to pseudoprogression, an increase in contrast tumor enhancement that mimics the true progression [3]. Therefore, it is crucial to identify biomarkers that may help establish the prognosis of patients with glioblastoma and predict their response to treatment.

Glioblastoma is a highly vascularized tumor that displays active angiogenesis [4]; thus, drugs with antiangiogenic properties, such as bevacizumab and cilengitide, are being tested for use with radiotherapy and temozolomide [5], [6]. Nevertheless, continuous temozolomide, as described for other metronomic chemotherapy regimens, might have antiangiogenic activity by itself, mediated in part by a direct effect on tumor vessel endothelium [7].

Circulating endothelial cells (CECs) consist of at least endothelial progenitor cells (EPCs) that originate in the bone marrow, mature endothelial cells shed from vessel walls, apoptic endothelial cells and some cells with endothelial function from cancerous cells [8]. Recently, circulating endothelial cells (CECs) have been established as markers of endothelial damage or dysfunction [9]. CEC levels increase in many kinds of disorders such as cardiovascular [10], [11], autoimmune [12], and infectious diseases [13], as well as in cancer [14]. In cancer patients, CEC number correlates with tumor progression [15] and constitutes a promising tool for monitoring disease activity, with potential for the assessment of prognosis and response to treatment. In patients with non-small cell lung cancer (NSCLC), we observed an association between elevated CEC numbers and decreased overall survival (OS) [16], although in a study by Kawaishi et al. [17], high CEC numbers were associated with longer progression-free survival (PFS). In patients with breast cancer treated with metronomic chemotherapy, CEC levels after two months treatment were associated with prolonged PFS [18]; in another trial with metronomic chemotherapy and bevacizumab, baseline CEC levels were also associated with PFS [19]. It has been suggested that quantification of CECs is useful to identify patients who might benefit from antiangiogenic treatments [20]. Batchelor et al., in a series of patients with glioblastoma treated with AZD2171, a pan-VEGF receptor tyrosine kinase inhibitor, found that viable CEC number increased when tumors escaped treatment [21].

Microparticles (MPs) are small vesicles (100 nm–1 µm) which directly bud from the plasma membrane of different cells, including blood, endothelial and tumor cells [22], [23]. During MP formation, phosphatidylserine (PS) is transferred from the inner to the outer leaflet of the membrane; this externalization of PS facilitates the assembly of components of the clotting cascade, thus increasing the procoagulant activity of MPs [24]. The procoagulant MP levels increase in cancer patients [25]. In patients with castration-resistant prostate cancer, high platelet-derived MP number is associated with shorter survival [26]. However, in patients with NSCLC we observed an association between elevated total MP count and increased OS [16]. Nevertheless, the potential prognostic value of MPs in glioblastoma patients remains unclear.

The aim of this study was to evaluate the potential prognostic value of CECs, MPs and MP-mediated procoagulant activity in patients with newly diagnosed glioblastoma. In addition, we have analyzed the methylation status of *MGMT* promoter in tumor tissue.

## Materials and Methods

### Study Design and Patients

This prospective study included consecutive patients with newly diagnosed, histologically proven glioblastoma who received standard treatment [1] at La Fe University Hospital. The control group comprised healthy subjects matched for sex and age with the patients. The study was conducted in accordance with the principles outlined in the Declaration of Helsinki. All participants gave written informed consent. The study was approved by the institutional Biomedical Research Ethics Committee.

After surgery, patients received radiotherapy to a total dose of 60 Gy in 30 fractions given five days per week, plus concomitant temozolomide at a daily dose of 75 mg/m^2^. After a four-week rest, adjuvant temozolomide was administered at a dose of 150 to 200 mg/m^2^ for five days every four weeks until progression, unaccepable toxicity or other reasons that hinder treatment. Patients were assessed by magnetic resonance imaging (MRI) at baseline. Subsequent imaging assessments were performed within 72 hours after surgery, to check the extent of tumor resection and to rule out postsurgical complications, and every three cycles of temozolomide thereafter. Perfusion, diffusion, and spectroscopy MRI procedures were performed when indicated. At progression, patients amenable for second-line treatment received bevacizumab plus irinotecan, fotemustine or rechallenge with temozolomide.

### Biomarker Evaluation

#### Blood sampling

Venous blood samples were obtained from patients within two weeks before the start of radiochemotherapy and during the last week of this treatment. The initial 3 mL of blood was discarded to avoid contamination with endothelial cells from the puncture wound of the vein. Blood for quantification of CECs was collected in a tube containing ethylenediaminetetraacetic acid (1.8 mg/mL). For the determination of MP levels and MP-mediated procoagulant activity, blood was collected in a tube containing sodium citrate (129 mM) at a ratio of 1∶9 (v/v, sodium citrate/blood). We have previously studied pre-analytical conditions to analyze MP count and pro-coagulant activity, and centrifugation at 1500×g, for 30 min, at 4°C and analysis on frozen plasma samples have been applied. Plasma was stored at –80°C to allow later batch analysis.

#### Quantification of circulating endothelial cells

The isolation and quantification of CECs was performed by immunomagnetic technique following a consensus protocol [27]. In brief, cells were isolated from whole blood at 4°C by means of an endothelial cell specific monoclonal antibody sEndo1 (BioCytex, Marseille, France) raised against the endothelial antigen CD146, coupled to micromagnetic beadsPan-Mouse M450 Dynabeads.Dynal, Oslo, Norway. To avoid nonspecific binding of leukocytes to CD146-coated beads, cells were incubated after immunomagnetic isolation of CECs with fluorescein isotiocyanate-conjugated (FITC)-*Ulex europaeus* lectin-1 (UEA1). UEA-1 lectin (Sigma-Aldrich, Inc., Saint Louis, MO, USA) is a good histologic marker for endothelium in human, and constitutes a specific and sensitive additional tool in demonstrating endothelial cells and endothelial derivation of human tumors. After incubation, samples were washed, suspended in buffer, and counted with fluorescence microscopy using a Nageotte chamber. The size of the CEC population often exceeds 10 µm, which is not compatible with the typical size of endothelial progenitor cells. In addition, the morphology of our cells indicates considerable damage or even necrosis. Nucleated cells >10 µm in length with more than eight immunomagnetic beads attached and positive UEA1 staining were regarded as CECs. Conglomerates were counted as one cell. The number of CECs was expressed as cells/mL of blood. Reproducibility was tested by performing six replicates of 10 different samples; the coefficient of variation was 12%.

#### Quantification of total microparticles

Plasma MPs were quantified by flow cytometry in an EPICS XL-cytometer (Beckman Coulter, Brea, CA, USA) at high flow rate. Plasma was incubated with FITC–Annexin V conjugate (TACS Annexin V; Trevigen Inc. Gaithersburg, MD, USA) to detect accessible phosphatidylserine on MP membranes. Standard fluorescent beads of different diameters were used for size calibration (0.5–3.0 µm, Megamix; BioCytex, Marseille, France) and to set the gate for MP detection at a diameter of 0.5–1 µm following a consensus guideline on MP measurement [28]. The number of FITC–Annexin V-positive MPs was calculated and expressed as events/µL of plasma.

### Assessment of MP-mediated Procoagulant Activity

The MP-mediated procoagulant activity of plasma was analyzed by thrombin generation (TG) assay without added exogenous tissue factor or phospholipids (Calibrated automated thrombogram, CAT; Thrombinoscope BV, Paris, FranceG). Under these conditions, the assay was critically dependent on MPs present in plasma. Curves were calculated using the Thrombinoscope software and the results were expressed as the thrombin peak (nM). MP activity depends on the exposure of anionic phospholipids that provide a surface for the assembly of the tenase and prothrombinase complexes. To measure this activity the procoagulant phospholipid-dependent clotting time (PPLCT) assay was also analyzed (STA-Procoag-PPL; Diagnostica Stago, Paris, France).

#### MGMT methylation analysis

Formalin-fixed, paraffin-embedded tumor samples were subjected to careful histological assessment in order to select tumor areas. Three non-tumor areas of brain tissue were further isolated and independently mounted in paraffin blocks. DNA from 10 µm sections of each tumor and from the three controls was extracted with the deparaffination solution (Qiagen, Venlo, The Netherlands), followed by its purification with the DNA Investigator kit (Qiagen). Methylation status of *MGMT* promoter was determined by multiplex ligation-dependent probe amplification (MLPA) following the manufacturer’s protocol (ME011-B1 MLPA, MRC-Holland). A methylation-sensitive restriction enzyme, HhaI (New England BioLabs), which cuts unmethylated GCGC sites, was applied to each set of samples. Reaction products were resolved on ABI3700 automated DNA sequencer and quantification of the methylation status of *MGMT* promoter was performed by Coffalyser software (MRC-Holland).

### Statistical Analysis

The Kolmogorov-Smirnov test was used to evaluate whether each parameter came from a normal distribution. Statistical analyses were performed using a non-parametric test among sample types (control, pre-treatment and post-treatment) (Krusal-Wallis test) and for the independent relationship of the control samples with respect to patient groups (Chi-Square test with Yate’s correction). Bivariate correlation was performed using Spearman’s correlation test. OS and PFS were analyzed by Kaplan-Meyer method and survival curves of subgroups were compared using the log-rank test. CEC values were dichotomized as greater than 99% confidence interval in healthy controls (CEC = 20 cells/ml). All statistical calculations were performed using SPSS software (v. 15.0; SPSS Inc., Chicago, IL, USA).

## Results

### Patient Characteristics

Twenty-two patients and forty healthy subjects were included in the study during a period of 18 months. Patients’ characteristics are shown in Table 1. All patients had histologically confirmed glioblastoma. Median PFS was 30 weeks (9–135) and median OS was 33 weeks (10–146).

**Table 1:** Clinical characteristics of patients.

Characteristics	Patients (n = 22)
Age in years	
Median (range)	62 (41–83)
Sex	
Male (%)	12 (54.5)
Female (%)	10 (45.5)
KPS score	
60–80 (%)	16 (72)
90–100 (%)	6 (27)
Extent of surgery	
S. Biopsy	5
Open biopsy	3
Partial resection	12
Complete resection	2

KPS: Karnofsky performance scale; S. Biopsy: stereotactic biopsy.

### Circulating Marker and Correlation Analysis

**Figure 1:**
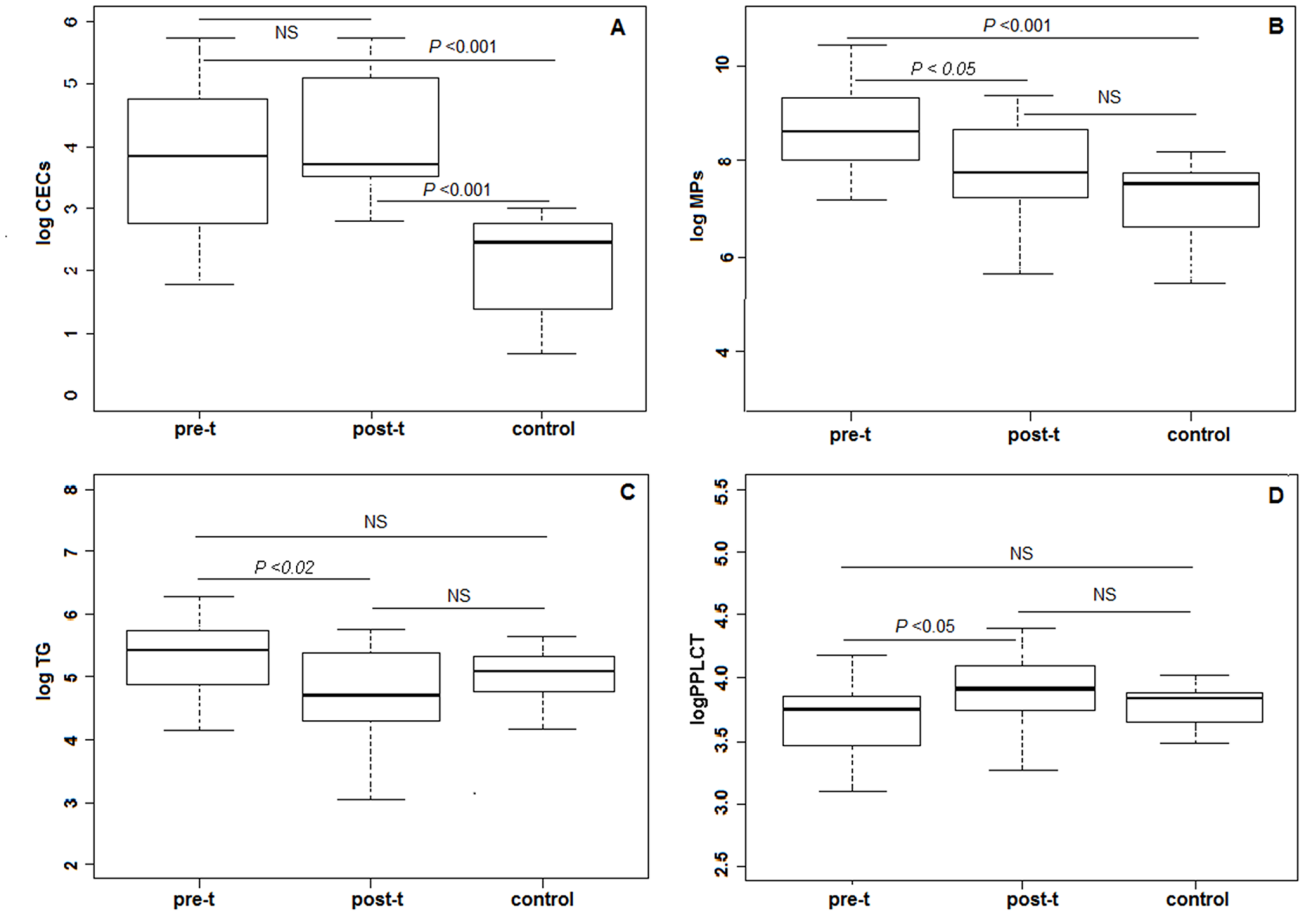
Pre- and posttreatment levels of biomarkers in patients and in controls. A: Circulating endothelial cells (CECs), B: Microparticles (MPs), C: Endogen thrombin generation (TG); D: Procoagulant phospholipid-dependent clotting time (PPLCT). Marker levels and their standard deviations are shown for pre-t: pretreatment; post-t: posttreatment; c: controls. Logarithmic transformation of data was made to normalize the distributions. NS: no significant.

Levels of CECs, MPs, TG and PPLCT in patients and controls are shown in Figure 1. Compared with the healthy control group, mean pre-treatment levels of CECs and MPs were significantly higher (p<0.001). Post-treatment levels of CECs remained significantly higher in patients than in controls, while levels of MPs and TG decreased, and PPLCT increased significantly after treatment. Significant correlations were found between both pre- and post-treatment levels of TG and MPs (p<0.01), while PPLCT inversely correlated with pre-treatment levels of MPs (p<0.01) and TG (p<0.05) (Table 2). Platelet and leukocyte count significantly decreased after treatment (platelet: 283 vs 166×10^3^, p<0.0001; leukocyte: 8.6 vs 6.2×10^3^, p = 0.021). Platelet count significantly correlated with TG (r = 0.52, p<0.001), MPs (r = 0.39, p = 0.013) and PPLCT (r = −0.49, p<0.01).

**Table 2:** Spearman’s bivariate correlation between MP and coagulation markers.

		MPs	PPLCT
**TG**	Pretreatment	0.732**	−0.627*
	Posttreatment	0.741**	
**PPLCT**	Pretreatment	−0.858**	
	Posttreatment		

MPs: circulating microparticles; TG: endogenous thrombin generation;

PPLCT: procoagulant phospholipid-dependent clotting time.

* p<0.05;

** p<0.01.

### Circulating Markers, *MGMT* Status and Clinical Outcome

**Figure 2:**
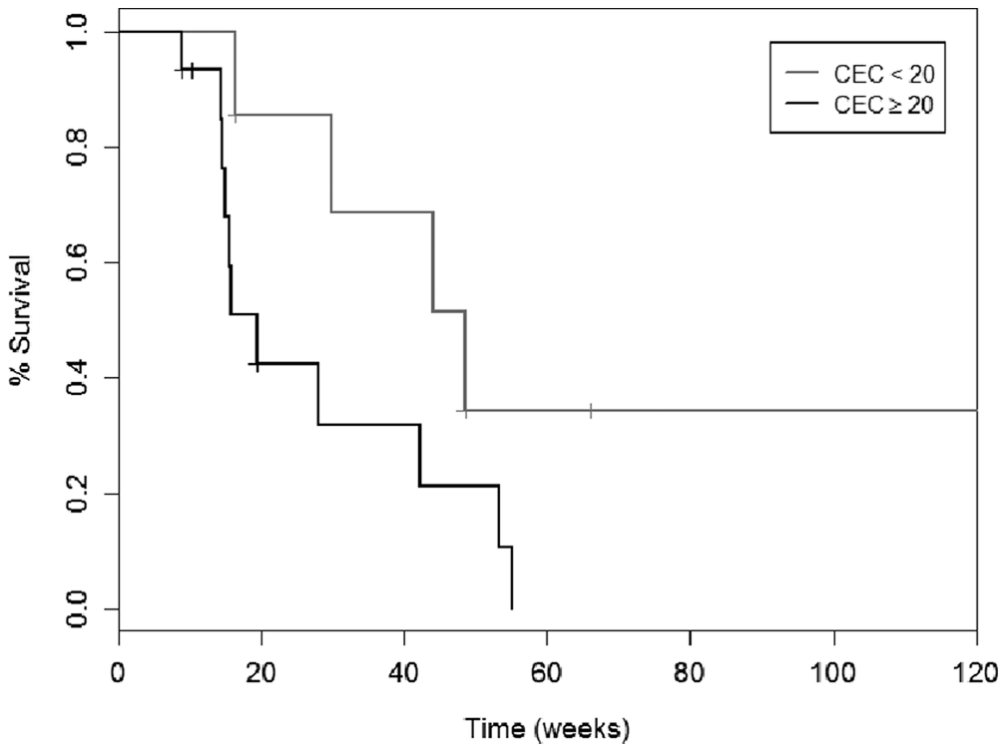
Kaplan-Meyer analysis of survival. Overall survival curve according to pretreatment CECs values were dichotomized as greater than 99% confidence interval in controls (CEC = 20 cells/ml) (Log rank = 4.566; p = 0.033). CECs: circulating endothelial cells.

The analysis of the associations between circulating markers and clinical outcome showed that pre-treatment CEC levels >20 cells/mL (corresponding to the 99^th^ percentile in controls) were associated with poor OS (19 vs. 72 weeks; Log rank 4.566; p = 0.033) (Figure 2). No such association was found for pre- or post-treatment levels of MPs, TG and PPLCT.

*MGMT* promoter was methylated in 27% of tumor samples. *MGMT* status significantly influenced median OS, which was 66 weeks (95% CI, 44.6 to 87.4 weeks) in *MGMT* promoter methylated patients and 30 weeks (95% CI, 14.4 to 45.5 weeks) in *MGMT* promoter unmethylated patients (P<0.004), although its influence on PFS was not statistically significant. The difference in OS observed according CEC pre-treatment levels and TG post-treatment levels did not reach statistical significance when analyzed separately in methylated and unmethylated patients.

## Discussion

Our results show that pre-treatment CEC levels were significantly elevated in patients with glioblastoma compared with controls, a finding that is consistent with those of previous studies in different types of cancer [14]–[16]. We have found an association between higher basal CEC count (>99^th^ percentile of the CEC count in controls) and poor survival. In other tumors, the association between baseline CEC count and clinical outcome is conflicting [15]–[17], [29]. Specific tumor characteristics and the variety of methods being used to identify CECs may explain these discrepancies. The standard treatment received by all patients in the present study includes low-dose, daily temozolomide along with radiation therapy. A study using a murine model reported that low-dose; continuous (metronomic) chemotherapy leads to apoptosis of endothelial cells within the tumor bed, resulting in increased apoptosis of tumor cells [30]. In patients with breast cancer receiving metronomic chemotherapy with methotrexate and cyclophosphamide with or without thalidomide, an increase in CEC count after two months was associated with a better PFS [18]. Similar results (i.e., an association between good outcome and an increase in CEC count after several treatment cycles) have been found in patients with cancer receiving antiangiogenic drugs [31], [32]. Nevertheless, a recent study in patients with colorectal cancer concluded that high viable CEC count both at baseline and after the first cycle of chemotherapy plus bevacizumab, was associated with a worse outcome [33]. In the present study, the CEC levels were higher than in controls; however, they did not increase significantly after radiochemotherapy. The impact of radiation therapy on CEC number has not been studied fully, and any effect could have influenced the post-treatment CEC count in our patients.

We found that the pretreatment MP count was significantly elevated in glioblastoma patients compared with controls. In agreement with our findings, microvesicles together with exosomes have also been reported to be elevated in glioblastoma patients and decrease upon temozolomide treatment [34].

Another finding of the present study is the decrease of MP and TG levels, and the increased PPLCT observed after treatment, an effect that could be explained by a decrease in the levels of their parent cells or by an inhibition of MP release induced by radiochemotherapy; although leukocytes and platelets decreased after treatment, only platelet count significantly correlated with TG, MPs and PPLCT. In a recent work by Sartori et al. [35], the procoagulant activity of annexin V-positive MPs was analyzed in 61 patients with glioblastoma at different times in their evolution; in accordance with the present study, they found that MP activity became significantly lower 1 and 4 months after surgery, though only in patients achieving complete surgical resection. As expected, in the present study OS was higher in patients with methylated *MGMT* promoter.

In summary, this exploratory study suggests an association between postsurgical higher CEC count and shorter survival in patients with glioblastoma. We believe that these findings warrant further investigation with a larger number of patients.
